# Association of screen time with long-term stress and temperament in preschoolers: results from the DAGIS study

**DOI:** 10.1007/s00431-020-03686-5

**Published:** 2020-05-27

**Authors:** Marja H. Leppänen, Katri Sääksjärvi, Henna Vepsäläinen, Carola Ray, Pauliina Hiltunen, Leena Koivusilta, Maijaliisa Erkkola, Nina Sajaniemi, Eva Roos

**Affiliations:** 1 Folkhälsan Research Center, Topeliuksenkatu 20, 00250 Helsinki, Finland; 2 Faculty of Medicine, Clinicum, University of Helsinki, PO BOX 21, 00014 Helsinki, Finland; 3 Faculty of Educational Sciences, University of Helsinki, PO BOX 8, 00014 Helsinki, Finland; 4 Department of Food and Nutrition, University of Helsinki, PO BOX 66, 00014 Helsinki, Finland; 5 Department of Social Research, University of Turku, Turku, Finland; 6 Philosophical Faculty, School of Applied Educational Science and Teacher Education, University of Eastern Finland, PO BOX 111, 80101 Joensuu, Finland; 7 Department of Public Health, Clinicum, University of Helsinki, PO BOX 20, 00014 Helsinki, Finland

**Keywords:** Hair cortisol concentration, Pediatrics, Television, Temperament

## Abstract

Screen time is increasing rapidly in young children. The aim of this study was to examine associations of long-term stress and temperament with screen time in Finnish preschool children and the moderating role of socioeconomic status. Cross-sectional DAGIS data were utilized. Long-term stress was assessed using hair cortisol concentration, indicating values of the past 2 months. Temperament was reported by the parents using the Children’s Behavior Questionnaire (the Very Short Form), and three broad temperament dimensions were constructed: surgency, negative affectivity, and effortful control. Screen time was reported by the parents over 7 days. The highest education level in the household was used as an indicator of socioeconomic status. In total, 779 children (mean age, 4.7 ± 0.9 years, 52% boys) were included in the study. Of the temperament dimensions, a higher effortful control was associated with less screen time (*B* = − 6.70, *p* = 0.002). There was no evidence for an association between hair cortisol concentration and screen time nor a moderating role of socioeconomic status in the associations (*p* > 0.05).

*Conclusion*: Our findings indicate that preschool children with a higher score in effortful control had less screen time. Because effortful control reflects general self-regulatory abilities, promoting these skills may be effective in reducing screen time in young children.

**Table:** 

**What is Known:** • *Screen time has increased rapidly during the last decades, and higher screen time has been linked with numerous adverse health consequences in children.* • *There are no previous studies investigating associations of long-term stress and temperament with screen time in young children.*	
**What is New:** • *Of the temperament dimensions, effortful control was associated with higher screen time in preschool children, but there was no association found between long-term stress and screen time.* • *Since effortful control reflects general self-regulatory abilities, promoting these skills may be effective in reducing screen time in young children.*

**What is Known:**

• *Screen time has increased rapidly during the last decades, and higher screen time has been linked with numerous adverse health consequences in children.*

• *There are no previous studies investigating associations of long-term stress and temperament with screen time in young children.*

**What is New:**

• *Of the temperament dimensions, effortful control was associated with higher screen time in preschool children, but there was no association found between long-term stress and screen time.*

• *Since effortful control reflects general self-regulatory abilities, promoting these skills may be effective in reducing screen time in young children.*

## Introduction

Screen time (ST), commonly divided as television (TV) viewing and the use of computers, mobile phones, or tablets, has increased rapidly since the 2000s [1]. It has been suggested to limit ST for children aged 2–5 years to 60 min/day [2], but only 24% of children met the recommendation in Canada in 2017 [3]. Moreover, in the UK in 2018, children aged 5 years had over 27 h a week of ST [4]. This is concerning because a higher ST has been connected with adverse health consequences, such as obesity and depressive symptoms in children [5]. Because health behaviors, including family ST, are established already in early childhood [2], it is essential to gain more knowledge of potential factors influencing it.

Traditionally, stress has been perceived as a part of adults’ lives [6], but during the last years, it has become more evident that stress is present already in young children’s everyday lives [7]. Young children are unreliable in reporting their symptoms; hence, the stress hormone cortisol can be used as an indicator of stress [8]. Cortisol is one of the end products of the hypothalamic–pituitary–adrenal (HPA) axis. Cortisol release occurs in a daily pattern, facilitating physiologic diurnal regulation [9, 10], and in bursts in response to stressors [11]. Hair cortisol concentration (HCC) is a relatively new method of assessing long-term cumulative cortisol levels, and it has been found to be a feasible tool for stress-related research [8, 12, 13]. A previous study in school-aged children reported that increased stress levels, as assessed by questionnaires, are connected to children’s health behaviors, such as decreased physical activity and increased sedentary behavior [14]. Furthermore, because watching TV has been found as one of the most frequently endorsed ways of coping with stress for school-aged children [15], there is a great need to study the association between long-term stress as assessed by HCC and ST in preschool children and fill the gap in the current literature.

Children develop in an environment that is a product of their characteristics and environmental factors [16]. For instance, children may be differentially sensitive to the effects of the environment depending on their temperament [17]. Temperament is often divided into three dimensions: (1) surgency (characterized, e.g., by high activity level and impulsivity), (2) effortful control (characterized, e.g., by inhibitory control and low-intensity pleasure), and (3) negative affectivity (characterized, e.g., by sadness, fear, and difficulty to soothe) [18]. It has been hypothesized that a child’s self-regulation (i.e., the capacity to engage in goal-directed behavior) may be linked to health behaviors [19]; therefore, it is essential to clarify relationship between temperament dimensions and ST to be better able to target health-promoting actions by taking child’s personal characteristics into account. Thus, the aims of the present study were to examine whether long-term stress assessed by HCC and/or temperament is associated with ST in a sample of Finnish preschoolers. Furthermore, because a higher socioeconomic status (SES) has been found to associate with less ST [20], we aimed to examine the moderating role of SES in the associations.

## Materials and methods

The present study utilizes cross-sectional data from the DAGIS study (the Increased Health and Wellbeing in Preschools Study), which aimed to diminish socioeconomic differences in preschool children’s energy balance-related behaviors [20]. The study was conducted in early childhood education and care (ECEC) centers in southern and western Finland in 2015–2016. The eligibility criteria for the study were as follows: (1) having at least one group consisting of 3–6-year-old children, (2) providing early education only during the daytime, (3) being Finnish or Swedish speaking (official languages of Finland), and (4) charging income-dependent fees. In total, 864 children (25% of the invited children, boys 52%) and their families from 66 ECEC centers (43% of the invited ECEC centers) in 8 municipalities agreed to participate in the study. Guardians gave their written informed consent. The study was approved by the University of Helsinki Ethical Review Board in the Humanities and Social and Behavioral Sciences in February 2015 (#6/2015).

Children’s age, gender, and the time spent in ECEC (hours/week) were reported by the parents. Weight and height were measured by trained researchers, and thereafter, body mass index (BMI) was calculated as body weight (kg)/height^2^ (m). The BMI standard deviation score (BMI-SDS) was computed by the national references [21]. The threshold for being overweight was defined using the age- and gender-specific BMI cut-offs of the International Obesity Task Force criteria [22].

The highest education level in the household was used as an indicator of SES. The educational level of both parents was inquired by a questionnaire, and the higher one was further categorized as lower than a bachelor’s degree (i.e., comprehensive, vocation, or high school), bachelor’s degree (i.e., bachelor’s degree or college), or higher than a bachelor’s degree (i.e., master’s degree or licentiate/doctor).

ST was reported by the parents using a 7-day diary. The diary was based on a previously validated diary [23], and it was further translated and modified into the Finnish context. Parents were asked to assess the frequency and time (hours/min) that their child spent each day: (1) watching TV, (2) watching DVDs or videos, (3) using tablets or smartphones, and (4) using computers or playing computer games. ST is a composition variable of all the above-mentioned types of ST. ST was calculated as follows: [(mean ST on weekdays × 5) + (mean ST on weekend days × 2)]/7.

Children’s temperament was evaluated using the Very Short Form of the Children’s Behavior Questionnaire that was developed for children aged 3–8 years [18]. One parent in each family indicated their opinion on the 36 items included in the questionnaire, using a 7-point Likert scale ranging from 1 (extremely untrue) to 7 (extremely true). Three broad temperament dimensions established by instrument developers were constructed from the questionnaire (12 items in each): surgency, negative affectivity, and effortful control. High levels of surgency refer to children who exhibit impulsivity, who enjoy situations with high stimulus intensity, and who do not show discomfort in social situations. Negative affectivity refers to children who typically have a lowered mood and are angry, fearful, and very difficult to soothe. Effortful control refers to children who have the capacity to suppress inappropriate responses, have better self-regulation, and can maintain focus on task-related activities [18]. Examples of the questions in each dimension have been previously published [24]. The questionnaire has been shown to demonstrate acceptable internal consistency and criterion validity in children [18]. In the present study, the Cronbach’s alpha values for surgency, negative affectivity, and effortful control were 0.80, 0.76, and 0.74, respectively.

Children’s long-term stress was assessed by HCC, which captures long-term integrated cortisol levels [25]. Trained preschool personnel collected hair samples from the posterior vertex of the scalp of the children. A hair lock of approximately 40 hairs was tied together and cut as close to the scalp as possible. The scalp end of the hair sample was marked, and the sample was packed in foil and put in a small plastic bag to send to a laboratory for analysis. In the laboratory, the strands were lined up and cut into two separate 2-cm segments. The laboratory followed the protocol of Davenport et al. [26] for the washing of hair and steroid extraction. A chemiluminescence immunoassay was used to measure the HCC from the hair samples (IBL, Hamburg, Germany). Both the intra- and inter-assay coefficients of variance (CV%) were less than 12%. Because boys had generally shorter hairs compared with girls, we used only the proximal 2-cm segment of the hair sample to include as many children as possible. In addition, it has been reported that ultraviolet radiation and hair care practices may decrease HCC levels [16], and it has been suggested to use a maximum 3-cm proximal hair segment [27]. Thus, the HCC (pg/mg) we report indicates stress approximately over the past 2 months. Because of the skewed distribution, the HCC was categorized into quintiles, and the first category (the lowest HCCs) was set as the reference group.

Descriptive information is given as arithmetic means or medians and standard deviations (SD) or frequencies and percentages (%). Gender comparisons among average values were made by using an independent t test or Mann Whitney U test for continuous variables and a chi-square test for categorized variables. ST had four outliers beyond a z-score of 3.29, and they were replaced using an equation (the mean plus two times standard deviations), in accordance with Field [28]. Using multiple linear regression, we examined the associations of ST with (1) long-term stress and (2) temperament in crude and adjusted models. Long-term stress was analyzed using HCC quintiles, and the first category (the lowest HCCs) was set as the reference group. Temperament was analyzed as continuous variable, and we included all three broad dimensions (surgency, negative affectivity, and effortful control) in the model simultaneously. Because SES has been related to HCC [27] and ST [20], we also investigated whether SES has a moderating role in the aforementioned associations. The differences in the average values of the log-transformed HCC, temperament dimensions, and ST between SES categories were examined using a one-way ANOVA and the differences between the categories using Tukey post hoc test. Each linear regression model was adjusted for the child’s gender (girl/boy), age, BMI, and time spent in ECEC (hours/week) due to their potential effect on the results. As a sensitivity analysis, we investigated long-term stress assessed as the mean of HCC from the two 2-cm segments. The results did not differ essentially, and thus, we decided to present the results using only the proximal 2-cm segment. We also tested whether gender is a modifier of the associations of long-term stress and/or temperament with ST. However, there was no evidence for gender-interactions between the studied variables, and therefore, the results were presented for boys and girls together. All statistical tests were conducted using the two-sided 5% level of significance and performed using SPSS Statistics 25 (IBM, Armonk, NY, USA). The moderating analyses were conducted with Hayes’s macro [29] for SPSS, version 3, using bootstrapping at the level of 10,000. The level for statistical significance for these analyses was set at *p* < 0.05.

## Results

All 779 children with complete data on ST (including ≥ 3 weekdays and ≥ 1 weekend day) were included in the current study (Table 1). Out of the 779 children, 631 (81%) had data in HCC and 697 (90%) in temperament. Children had ST on average 76 (± 35.8) min/day, their median HCC was 11.8 (range 0.18–808) pg/mg, the scores for temperament dimension surgency was on average 4.7 (± 0.8), the scores for negative affectivity was 3.7 (± 0.9), and the scores for effortful control was 5.2 (± 0.7). Furthermore, boys were taller and heavier and had higher HCC as well as had higher scores for surgency and lower scores for effortful control compared with girls (Table 1). Compared with the children that were excluded from the current study, the participating children spent more time in ECEC (t test: *p* = 0.029), and their parents were more often highly educated (having at least a bachelor’s degree education) (chi-square test: *p* < 0.001).

**Table 1:** Descriptive characteristics of children

	All		Boys		Girls		
	*N*	mean ± SD	*N*	Mean ± SD	*N*	Mean ± SD	*p*^*d*^
Age (years)	779	4.7 ± 0.9	402	4.8 ± 0.9	377	4.7 ± 0.9	0.31
Height (cm)	743	109.6 ± 7.8	377	110.6 ± 7.8	366	108.6 ± 7.6	*<0.001*
Weight (kg)	741	19.2 ± 3.5	376	19.5 ± 3.5	365	18.8 ± 3.5	*0.005*
BMI-SDS^a^ (kg/m^2^)	742	− 0.04 ± 0.99	377	− 0.04 ± 0.98	365	− 0.04 ± 0.99	0.98
Overweight or obese^b^ (*N*, %)	742	86 (11.6)	377	40 (10.6)	365	46 (12.6)	0.40
Parental education level^c^ (N, %)	775		402		373		0.13
< Bachelor’s degree		163 (21.0)		84 (20.9)		79 (21.2)	
Bachelor’s degree		330 (42.6)		159 (39.6)		171 (45.8)	
> Bachelor’s degree		282 (36.4)		159 (38.9)		123 (33.0)	
Time spent in ECEC (h/week)	717	34.8 ± 8.7	371	35.1 ± 8.5	346	34.4 ± 8.9	0.27
Screen time (min/day)	779	75.9 ± 35.8	402	77.4 ± 36.6	377	74.2 ± 34.9	0.22
HCC (pg/mg) (median, range)	631	11.8 (0.18–808)	279	15.7 (0.28–347)	352	8.89 (0.18–808)	*< 0.001*
Temperament (7-point Likert scale)							
Surgency	697	4.7 ± 0.8	356	4.8 ± 0.8	341	4.6 ± 0.9	*0.008*
Negative affectivity	697	3.7 ± 0.9	356	3.7 ± 0.8	341	3.7 ± 0.9	0.50
Effortful control	697	5.2 ± 0.7	356	5.0 ± 0.7	341	5.4 ± 0.7	*< 0.001*

Differences with *p* < 0.05 were considered statistically significant.

*BMI-SDS* body mass index standard deviation score, *ECEC* early childhood education and care, *HCC* hair cortisol concentration, *SD* standard deviation

^a^According to Saari A, Sankilampi U, Hannila M, Kiviniemi V, Kesseli K, Dunkel L (2011) New Finnish growth references for children and adolescents aged 0 to 20 years: Length/height-for-age, weight-for-length/height, and body mass index-for-age. Ann Med 43(3):235–248

^b^According to Cole TJ, Lobstein T (2012) Extended international (IOTF) body mass index cut-offs for thinness, overweight and obesity. Pediatr Obes 7(4):284–294

^c^Lower than bachelor’s degree includes comprehensive, vocational, or high school; bachelor’s degree includes bachelor’s degree or college; and higher than bachelor’s degree includes master’s degree or licentiate/doctorate

^d^T test or Mann-Whitney *U* test for continuous variables; chi-square for categorized variables

In the unadjusted model, a one unit increase in effortful control was associated with over 4 min less ST per day (*p* = 0.026) (Table 2). Moreover, after adjusting for confounders, the association became stronger (*B* = − 6.70, *p* = 0.002). With regard to negative affectivity, a one-unit increase was associated with over 3 min more ST per day in unadjusted models (*p* = 0.035), but after adjusting for confounders, the association became non-significant. The associations of surgency or HCC with ST were non-significant (Table 2).

**Table 2:** Linear regression analysis of associations of long-term stress and temperament with screen time

	Screen time (min/day)				Screen time (min/day)			
	*N*	*R*^2^	Unadjusted *B* (95% CI)	*p*	*N*	*R*^2^	Adjusted^a^ *B* (95% CI)	*p*
1) Long-term stress	631	0.009			556	0.046		
First quintile			1.00				1.00	
Second quintile			0.90 (− 7.95 to 9.75)	0.84			2.18 (− 7.21 to 11.57)	0.65
Third quintile			4.38 (− 4.44 to 13.20)	0.33			2.55 (− 6.80 to 11.89)	0.59
Fourth quintile			7.67 (− 1.22 to 16.55)	0.091			5.42 (− 4.22 to 15.06)	0.27
Fifth quintile			− 1.90 (− 10.68 to 6.89)	0.67			1.27 (− 8.27 to 10.81)	0.79
2) Temperament	697	0.017			627	0.051		
Effortful control			− 4.29 (− 8.05 to − 0.53)	0.026			− 6.70 (− 10.85 to − 2.55)	0.002
Negative affectivity			3.32 (0.23 to 6.41)	0.035			1.38 (− 1.90 to 4.67)	0.41
Surgency			1.79 (− 1.49 to 5.07)	0.29			0.28 (− 3.20 to 3.76)	0.88

Values are *R* square, unstandardized *B* coefficients (95% confidence intervals), and *p* values. The *B* coefficients given in the table provide estimates of the change in screen time (min/day): (1) different quintiles compared with the first quintile in long-term stress and (2) associated with a one-unit difference in temperament dimensions

^a^Adjusted for age, gender, BMI, and hours spent in ECEC per week. In the models regarding temperament, all three temperament dimensions were entered to the model simultaneously. Statistically significant results are italicized

A post hoc Tukey test showed that there were differences between SES categories in the mean values in negative affectivity (low SES 3.97 versus middle SES 3.64, *p* < 0.001; low SES 3.97 versus high SES 3.59, *p* < 0.001) and ST (low SES 83.4 versus high SES 70.7, *p* = 0.001). HCC, surgency, or effortful control did not differ in terms of SES (*p* > 0.05), respectively. We also tested the moderator effect of SES in the associations of HCC or temperament dimensions with ST. After adjustments, there were no significant moderator effects found (all interaction terms *p* > 0.05).

## Discussion

Of the temperament dimensions, a higher score in effortful control was associated with less ST. Because effortful control has been linked to the capacity to suppress inappropriate responses, have better self-regulation skills, and the ability to maintain focus on task-related activities [18], the finding is somewhat expected. Thus, our results indicate that increased knowledge about associations of temperament dimensions with ST is essential when promoting children’s health. However, we did not find an association between long-term stress as assessed using HCC and ST nor the moderating role of SES.

To date, there is a lack of studies examining the association between temperament dimensions and ST in children; therefore, comparing our results with the others is difficult. Howe et al. [30] studied 2-year-olds, and they found no significant association between temperament and ST. However, they assessed temperament using the 30-item Colorado Childhood Temperament Inventor, which divides temperament into six dimensions. The different approaches to divide temperament dimensions may lead to contrary findings. Munzer et al. [31] studied 4.5-year-olds, and they reported that more ST was associated with a poorer self-regulation. Because self-regulation has been generally referred to as an ability to control one’s thoughts, feelings, and behaviors to achieve a goal [32], their finding is in line with ours. Furthermore, as discussed in their study, the association may be bidirectional [31], and this may also be the case in our study. It is possible that the children with higher scores in effortful control and who had less ST are better able to follow the parental rules for ST because of their temperament. Nevertheless, future studies using a longitudinal design to elucidate this association are still needed to be better able to support children’s health behaviors taking different temperaments into account.

We found no significant association between long-term stress as assessed using HCC and ST in preschool children. Previously, it has been reported that higher levels of stressors as assessed using child reports were associated with more sedentary behavior [14], but to the best of our knowledge, there are no studies using objective measures to assess stress in children. A study in adult women (*n* = 72), however, reported a non-significant association between HCC and TV viewing or computer use [33], which is similar to our study. Moreover, we did not find any moderator effect of SES. One explanation for these findings may be that the children had somewhat less ST than has previously been reported in preschool children in the literature (1.2 compared with 2.0–2.6 h per day) [3, 31, 34].

The public health significance of the findings also needs to be addressed. The children with a higher score in effortful control had 6.7 min/day less ST indicating a cumulative decrease of 47 min/week. Since ST has been noted the most prevalent leisure-time sedentary behavior in children [35] and ST has also been used as a proxy for sedentary behavior [36], it is possible that the health benefits related to the decrease in ST may not be limited only to the decrease in sedentary time but also increase in physical activity. This is noteworthy since physical activity in young children has been linked with numerous health outcomes [37]. Similarly, this has been noted in the study by McVey et al. [36] who reported that decrease in sedentary time and increase in physical activity may be essential in promoting bone health. Therefore, we may conclude that the health benefits from decrease in ST may occur due to both less sedentary time and more physical activity.

## Strengths and limitations

The strengths of the current study include a relatively large sample of children, objective assessment of long-term stress, and validated assessment of temperament. Although assessing HCC in young children is somewhat new, it has been recognized as a valuable tool in research [8, 27]. The use of daily ST diaries with open questions instead of ready-given response categories in assessing ST was chosen to increase the representativeness of ST. Furthermore, the daily ST diary included all types of ST (i.e., watching TV, watching DVDs or videos, using tablets or smartphones, and using computers or playing computer games) instead of restricting it only to TV viewing [5].

The study also has some limitations that need to be considered. Firstly, HCC is an indirect indicator of stress, and it assesses all exposure to cortisol. As has been stated, HCC has been found to be elevated in children from low SES families but also in children who have perceived poorer temperament or behavior (i.e., are more fearful or have socioemotional issues) [8]. Thus, research is still needed to clarify the role of the developing HPA axis in the level of HCC as well as the role of elevated HCC in response to potential stress exposures in young children. We are not aware of the children’s medication use; therefore, we could not take it into account in the analyses. On the other hand, recent literature has shown contrary results about the role of medication in HCC levels when the studies have used small samples sizes (*n* = 18–108) [27]. Likewise, we did not have information about hair-wash frequency, which has been considered as a possible confounding factor in HCC research [12]. However, in a review of children [27], there was no evidence found for the need to take hair-wash frequency into account. The cross-sectional study design limits the conclusion about causality between the observed associations. However, because HCC indicated long-term stress over the past 2 months, we can speculate that long-term stress was predicting ST and not vice versa. Finally, because of the relatively low participation rate, the sample in our study may be somewhat selected. It is possible that the families with lower SES declined to participate. Because SES has been negatively related to HCC [27] and ST [20], the HCC levels in the current study may have been lower than in the general population. Furthermore, we cannot exclude the possibility that the higher SES families may have underreported their child’s ST because of their increased awareness about suitable ST limitations.

In future studies, there is a need to investigate associations of different types of ST (e.g., is the use passive versus active, is the use for entertaining versus educational purposes, or is she/he watching stationary device versus playing with a touchscreen device) with HCC and temperament. In addition, impact of different types of ST in the adverse health consequences should be further investigated. For instance, as Chindamo et al. [38] have reported, the use of tablets and smartphones was associated with poor sleep in toddlers, irrespective of the children’s temperament or viewing television. Their findings highlight the need to clarify more deeply the role of different types of ST for children’s health and development. Such knowledge would be beneficial for parents and child care personnel but also for the technology when developing solutions that can help to diminish ST-based adverse health consequences in the future.

In conclusion, of the temperament dimensions, a higher score in effortful control was associated with less ST. This information may be essential when planning interventions to reduce ST in preschool children. We did not find evidence of an association with long-term stress as assessed using HCC and ST nor the moderating role of SES. However, we believe this study will create a base for future studies in clarifying the role of long-term stress as assessed using HCC and/or temperament with ST but also with other health behaviors, such as physical activity and sleep, in young children.

## Abbreviations

BMI: Body mass index
BMI-SDS: BMI standard deviation score
CI: Confidence interval
ECEC: Early childhood education and care
HCC: Hair cortisol concentration
HPA: Hypothalamic–pituitary–adrenal
SD: Standard deviation
SES: Socioeconomic status
ST: Screen time
